# Multi-UAV Reconnaissance Task Assignment for Heterogeneous Targets Based on Modified Symbiotic Organisms Search Algorithm

**DOI:** 10.3390/s19030734

**Published:** 2019-02-12

**Authors:** Hao-Xiang Chen, Ying Nan, Yi Yang

**Affiliations:** Nanjing University of Aeronautics and Astronautics, Nanjing 210016, China; nanying@nuaa.edu.cn (Y.N.); nuaa_yang@nuaa.edu.cn (Y.Y.)

**Keywords:** UAV, reconnaissance task assignment, sensor, Pareto dominance determination, symbiotic organisms search

## Abstract

This paper considers a reconnaissance task assignment problem for multiple unmanned aerial vehicles (UAVs) with different sensor capacities. A modified Multi-Objective Symbiotic Organisms Search algorithm (MOSOS) is adopted to optimize UAVs’ task sequence. A time-window based task model is built for heterogeneous targets. Then, the basic task assignment problem is formulated as a Multiple Time-Window based Dubins Travelling Salesmen Problem (MTWDTSP). Double-chain encoding rules and several criteria are established for the task assignment problem under logical and physical constraints. Pareto dominance determination and global adaptive scaling factors is introduced to improve the performance of original MOSOS. Numerical simulation and Monte-Carlo simulation results for the task assignment problem are also presented in this paper, whereas comparisons with non-dominated sorting genetic algorithm (NSGA-II) and original MOSOS are made to verify the superiority of the proposed method. The simulation results demonstrate that modified SOS outperforms the original MOSOS and NSGA-II in terms of optimality and efficiency of the assignment results in MTWDTSP.

## 1. Introduction

Unmanned aerial vehicles (UAVs) have played increasingly important roles in military reconnaissance during the last decade. When dealing with multiple reconnaissance tasks [1], it is often required that UAVs form teams and work collaboratively, which necessitates cooperative task assignments [2]. The purpose of cooperative task assignments is to allocate necessary tasks and determine the appropriate task execution sequence to the UAVs to maximize overall performance. The battlefield situation and the range of permissible performance of the UAV set are commonly considered in the cooperative task assignments. 

The cooperative task assignment problem (CTAP) is usually considered as NP-Hard Problems. In some prior works, the basic CTAP was formulated as a mixed-integrated linear programming (MILP) problem [3], which aims to obtain an optimal assignment solution. Decentralized approaches were taken to deal with the dynamic, unexpected situations by using the information obtained by itself or through communications with neighboring UAVs [4]. With the intention of reducing the computational burden, optimization algorithms such as genetic algorithms (GA) were adopted to obtain suboptimal solutions [5]. Shaferman et al. [6] took UAVs’ dynamic constrains into consideration to ensure flyable trajectories and applied a stochastic search method to improve assignment solutions. Gyeongtaek et al. [7] put forward a market-based distributed assignment algorithm for timing constrained tasks and determine the allocation sequence by the estimated time of arrival (ETA) of each UAV. In the works mentioned above, UAVs’ paths to the task positions were not precisely calculated during the process of assignment. 

In the actual assignment planner, the path for the UAVs to perform the tasks should also be taken into consideration. Thus, in some other prior studies, the basic CTAP were formulated as a travelling salesman problem (TSP) [8], which aims to find the optimal scheme for a salesman to visit all the cities with the shortest path. For the original TSP, the length between two cities is usually considered as Euclidean length. According to the dynamic characteristics and constraints of UAVs, Dubins vehicle model and Dubins path were introduced into the CTAP for route length computation [9]. Thus, the CTAP was formulated as a Dubins travelling salesman problem (DTSP) [10]. Furthermore, Zhang et al. [11] took the effective range of UAVs’ sensors into consideration and formulated the CTAP as a Dubins travelling salesman problem with neighborhood (DTSPN). Wang et al. [12] considered the multi-UAV reconnaissance task allocation as an extended multiple DTSP, where the visit paths to the heterogeneous targets must meet specific constraints due to the targets’ feature. Since the reconnaissance tasks are time-sensitive, the execution time required will affect UAVs’ choices of tasks and flight paths. Considering the time windows of the tasks (targets), Karabulut et al. [13] formulated the TSP with a service time containing ready time and due time, which was so called TSPTW. Nunes et al. [14] explained the different circumstance, under which the time window was took as a hard temporal constraint and a soft temporal constraint. 

As for the subject of CTAP, the literature mentioned above concentrated on the heterogeneity features and kinematic constraints of UAVs, but the features of targets were ignored or considered to be homogeneous. In this paper, we focus on the reconnaissance task allocation problem for UAVs, considering ground targets with heterogeneous features and sizes are considered. To describe the CTAP, a novel cooperative reconnaissance task assignment model for multi-UAV is formulated: multiple time window-based Dubins travelling salesmen problem (MTWDTSP). Compared with the MILP form, MTWDTSP takes into account the influence the influence of various flight paths on the assignment results; as for compared with common TSP form, MTWDTSP could deal with the effect of different time windows on task sequence. In this particular problem, heterogeneous reconnaissance targets are also presented as point, strip, and surface targets, according to the features of targets and the performance of UAV sensor. To accomplish the reconnaissance of each target, UAVs must cover all the heterogeneous targets with the airborne sensors. 

The MTWDTSP is a typical NP-hard multi-objective optimization problem. Group intelligent optimization methods (such as Bayesian approach [15]) are employed to solve the problem with independent task points in a static environment within limited space, which can reduce the complexity of modeling and calculation. In recent years, heuristic and bio-inspired algorithms based group intelligent optimization methods have been adopted to solve the multi-objective optimization problem, including the simulated annealing algorithm [16], the Tabu search algorithm [17], genetic algorithms (NSGA-II) [18,19], the artificial fish school algorithm [20], ant colony algorithm [21] and the particle swarm optimization (niching PSO) [22,23,24,25]. These algorithms perform better than most traditional mathematical techniques in solving these problems, because they do not require substantial gradient information. Traditional group intelligent optimization algorithms can obtain locally optimal solutions for low-dimensional problems. As the number of UAVs and tasks (targets) grows, the traditional group intelligent optimization algorithms can easily premature and fall into local optimum. In order to search over the designed possible solution spaces as much as possible, Cheng and Prayogo [26] first proposed in 2014 a new meta-heuristic optimization algorithm known as symbiotic organisms search (SOS). In this paper, we introduce a modified multi-objective symbiotic organisms search (MOSOS) [27] to solve the MTWDTSP with dynamic time window constraints, task type constraints, and UAV sensor constraints. To improve the performance of MOSOS, a globally adaptive parasitism parameter [28] is used to increase convergence speed. Pareto optimal solution set is introduced to improve the variety of population and increase the probability of converging to the optimal solution. Also, a double-chain chromosomes encoding is adopted to pre-process the designed solution space for the efficiency of the algorithm. 

## 2. Mathematical Model of the MTWDTSP

In this section, the models of sensors, targets, and UAVs are established. The paths of covering targets with different shapes are also designed to calculate the estimated reconnaissance time.

### 2.1. Modeling of the UAV and Sensor

In the particular problem, a set of heterogeneous targets need to be reconnoitered by different types of UAVs subject to several types of constraints. In this article, Dubins model for UAV [10] is introduced with the following basic assumptions: (1) the velocity of each UAV is constant; (2) the UAVs perform the reconnaissance task at given altitudes; (3) the UAVs fly at different altitudes without inter-UAV collision; (4) the flight time of each UAV is limited. At this point, the spatial dimension of the UAV can be reduced from three dimensions to two dimensions (*X_U_-Z_U_* plane):

(1) $$S_{U}=(x,z,\psi ,V_{U},r_{\min})$$

(2) $$\{\begin{array}{c} \dot{x}=V_{U}\cos\psi \\ \dot{z}=V_{U}\sin\psi \\ \dot{\psi }=c\cdot V_{U}/r_{\min} \end{array}$$

where *x* and *z* represent the UAV’s coordinates in plane space, *V_U_* represents for the speed, *r_min_* represents the minimum turning radius, and ψ is the flight yaw angle.

**Figure 1:**
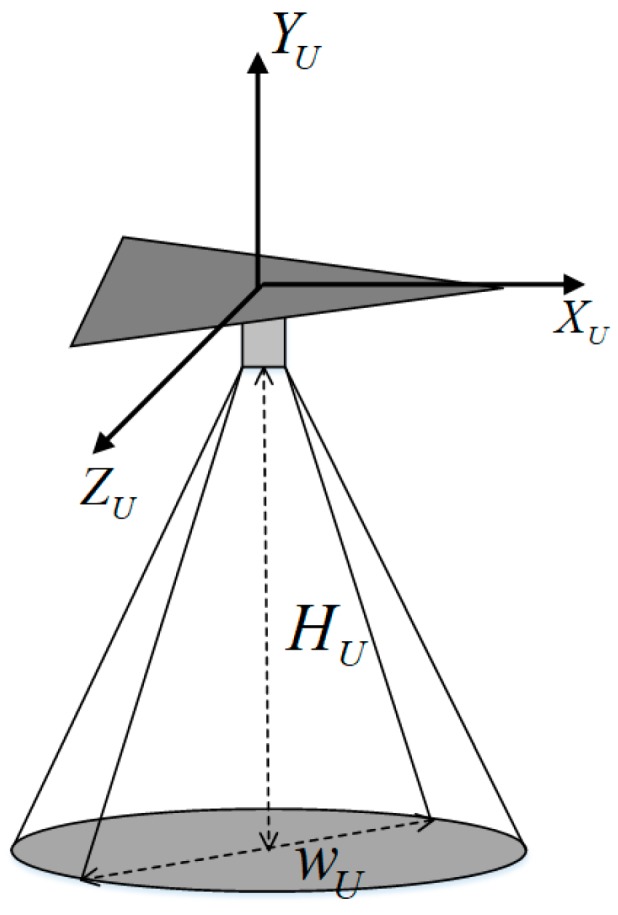
Body/dynamic system and airborne sensor of UAV.

Airborne sensors are required to reconnoiter ground targets. It is assumed that the reconnaissance task on this target is finished when a ground target is fully covered by the sensor’s field of view. In this study, a sensor’s field of view is considered as a circle region with the constant opening angle on the ground, neglecting the influence of altitude and noise, as illustrated in Figure 1. In Figure 1, *H_U_* is the flight altitude, *W_U_* is the detection width of the sensor, the axis *X_U_-Z_U_-Y_U_* denotes the body/dynamic system of the UAV. 

### 2.2. Modeling of Heterogeneous Ground Targets

**Figure 2:**
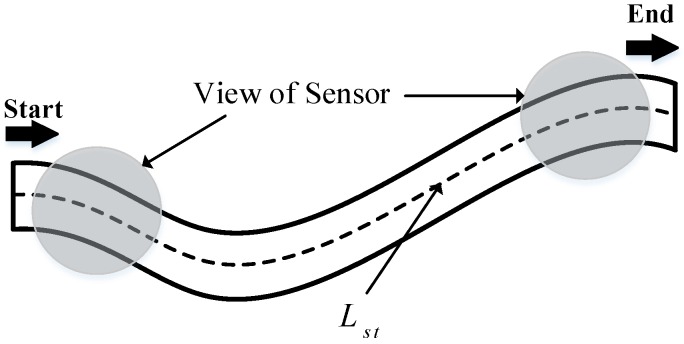
Reconnaissance of strip targets.

**Figure 3:**
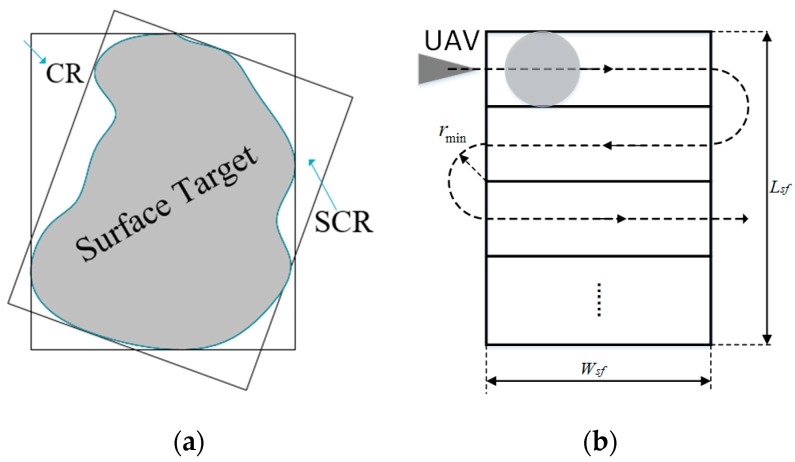
Reconnaissance of surface targets. (**a**) Smallest circumscribed rectangle of surface targets; (**b**) Reconnaissance path.

In general, one of the fundamental features is the target shape. To facilitate the determination of the reconnaissance duration and path, the shape of a target can be divided into point targets, strip targets, and surface targets. Due to the different sizes of targets, the same UAV will have different detection times for each type of target. Assuming that the detection time is always lower than the time window, then the following provisions are made to determine a suitable target detection time:

(1) For point targets, the default detection time is a fixed duration: $t_{d}=t_{Dec}$;
(2) For strip targets, the UAV detects targets along the direction of strip extension (shown as Figure 2), and the detection duration is $t_{d}=L_{st}/V_{U}$, where $L_{st}$ represents the extension length of the target and $V_{U}$ is the UAV’s speed.
(3) For surface targets, the UAV must fly along the “Z” path to detect the entire area. Considering the irregularity of the detection area, as shown in Figure 3a, an equal interval rotation method should first be performed to find the smallest circumscribed rectangle (SCR) of the irregular area. Then the circumscribed rectangle should be divided into several smaller rectangles, and finally, the UAV can detect the whole area along the “Z” path.
  Denote the SCR as $H_{min}=L_{sf}*W_{sf}$, where *L_sf_* and *W_sf_* represent the length and width, respectively, shown in Figure 3b. Therefore, the detection duration of surface targets is $t_{d}=\frac{1}{V_{U}}\left(L_{sf}\cdot \left\lceil W_{sf}/w_{U}\right\rceil +\pi w_{U}\cdot \left\lfloor W_{sf}/w_{U}\right\rfloor \right)$, where $w_{U}$ is the detection width of a single UAV, ⌈⋅⌉,⌊⋅⌋ represent for the upward and downward rounding operators.

It is worth pointing out that the way to select the entry point of the surface or the strip and the generation of Dubins-Path may be found in [12].

**Figure 4:**
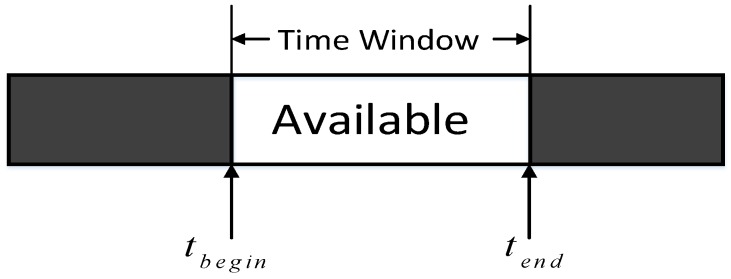
Time window of the reconnaissance task.

**Figure 5:**
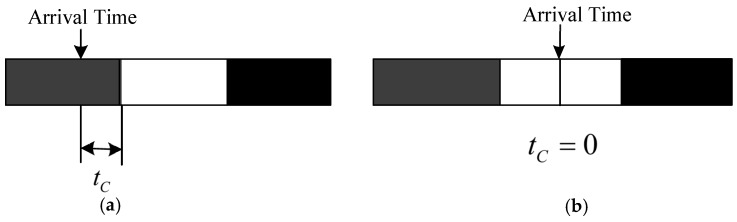
Schematic diagram of the waiting time and arrival time. (**a**) Arrive before available; (**b**) Arrive when available.

The second feature of heterogeneous target is the detectable time. The time attribute of the reconnaissance task means that the target has a fixed time window (as shown in Figure 4): $T_{task}=(t_{begin},t_{end})$, where $t_{begin}$ is the earliest time available for detection and $t_{end}$ is the latest time available, which is considered as a hard-constraint. Denote $Get_{uav}$ as the arrival time of the UAV, and then the waiting time (shown in Figure 5.) before execution $t_{c}$ can be calculated as follows:

(3) $$t_{C}=\left\{ \begin{array}{cc} t_{begin}-\;Get_{uav} & if\;t_{begin}>\;\;Get_{UAV}\\ 0 & if\;t_{begin}\leq Get_{UAV}<t_{end}\\ -1 & else \end{array}\right.$$

where $t_{c}=-1\;$ means the failure of this task.

### 2.3. Assignment Model of Reconnaissance Tasks for Multi-UAVs

Task assignment problem for multiple cooperative UAVs can be described as follows: for $N_{U}$ UAVs and $N_{T}$ reconnaissance tasks of geographically dispersed targets, under the decision variable ${Χ|}_{N_{U}}^{N_{T}}$, the whole group of the UAVs is able to complete all tasks satisfying all types of constraints $C_{Asn}(c_{1},c_{2}\cdots c_{N_{C}})$ while ensuring that the overall performance indicator $J_{Asn}$ will be optimized. The objective of the task assignment is to minimize the total cost (performance indicator), which can be expressed as:

(4) $$\{\begin{array}{c} {Χ|}_{N_{U}}^{N_{T}}=\arg\underset{X}{\min}(J_{Asn})\\ C_{Asn}(c_{1},c_{2}\cdots c_{N_{C}})\leq 0 \end{array}$$

Considering the reconnaissance model, the number of the UAVs, the type of restrictions, and the time window of the task itself, certain tasks may not be successfully implemented. Therefore, determining how to prioritize the important tasks and make the total flight distance as short as possible become the optimization goals of the problem. To describe the above progress in a mathematical form, the decision variable of task assignments can be given as follows:

(5a) $${X|}_{N_{U}}^{N_{T}}=\left\{\chi_{ij}^{kp}|i=1,2\cdots N_{U};j\in T;k=1,2\cdots N_{S};p=1,2\cdots N_{P}\right\}$$

(5b) $$\chi_{ij}^{kp}=\{\begin{array}{cc} 1, & UAV\;i\;for\;Task\;j\\ 0, & else \end{array}$$

where **T** = < 1,2….$\;N_{T}$> denotes the set of the number for task points, *k* denotes the type of UAV, *p* denotes the *p*th drone of the *k*th type, and the number of all reconnaissance tasks is $N_{T}$. Therefore, the decision variable has the following matrix form:

(6) $${X|}_{N_{U}}^{N_{T}}=\left[\begin{array}{cccccc} a_{00} & a_{01} & \cdots & \cdots & \cdots & a_{0T_{\max}}\\ a_{10} & a_{11} & \cdots & \cdots & \cdots & a_{1T_{\max}}\\ \vdots & \vdots & \ddots & & & \vdots \\ \vdots & \vdots & & \ddots & & \vdots \\ \vdots & \vdots & & & \ddots & \vdots \\ a_{T_{\max}0} & a_{T_{\max}1} & \cdots & \cdots & \cdots & a_{T_{\max}T_{\max}} \end{array}\right]=\left[\begin{array}{cccccc} 0 & 0 & \cdots & 1 & \cdots & 0\\ 0 & 0 & \cdots & \cdots & 1 & 0\\ \vdots & \vdots & \ddots & & & \vdots \\ 1 & \vdots & & 0 & & \vdots \\ \vdots & \vdots & & & \ddots & \vdots \\ 0 & 0 & 1 & \cdots & \cdots & 0 \end{array}\right]$$

Given that the base node number is 0, it can be seen that the element has the form of a sparse matrix. In this study, considering that each task reconnoiters only once at most, there is at most one nonzero item per row in the sparse matrix.

### 2.4. Mathematical Model of the Performance Indicator (PI) and Constraints 

In this problem, the constraints of assignment involve two parts: physical and logical. Physical constraints are related to UAVs’ flight characteristics and task properties, while logical constraints focus on the requirements of each type of task and the strategies for UAVs to implement the reconnaissance tasks. Some of the fundamental constraints are as follows [29,30]:

(1) All UAVs start from the base and eventually return to the base.
(2) Each task can be completed no more than once.
(3) The assignment scheme should match the time window and imaging requirements of each individual task.
(4) Each UAV can be assigned only one task at the same time period.
(5) The total time for each UAV to implement the tasks should not be longer than the longest flight time.

The details of the constraints are described as below:

(1) PIs based on task assignment:

(7) $$\min J_{1}(Χ)=\lambda (N_{U}+2-S(Χ))+(1-\lambda )N(Χ)$$

(8) $$S\left(Χ\right)=\;\overset{N_{k}}{\underset{k=1}{\sum }}\overset{N_{p}}{\underset{p=1}{\sum }}\sum_{i=1}^{N_{U}}\overset{N_{T}}{\underset{j=1}{\sum }}w_{j}\chi_{ij}^{kp}$$

(9) $$N_{U}=\;\overset{N_{s}}{\underset{k=1}{\sum }}N_{p}$$

where S(X)  represents the total weight of the tasks that have been assigned, $w_{j}\;$ reflects the importance of the *j*th reconnaissance task, $N_{U}$ is the total number of all UAVs, and N(X) is the number of the employed UAVs to execute the assigned tasks. 

The PI (7) is designed in a flexible form with the purpose to let the task assignment model choose the proper objective function to optimize depending on the conditions of UAVs and tasks. λ represents the adjustment parameter, and divides $min\;J_{1}\left(X\right)$ into two parts: when λ=1, $\min J_{1}(Χ)=\min(N_{U}+2-S(Χ))$, which indicates that the PI chooses to optimize the maximum weight of assigned tasks; when λ=0, $\min J_{1}(Χ)=\min N(Χ)$, which indicates that the PI chooses to optimize the minimum number of the employed UAVs. 

Defining the symbolic functions as the following form:

(10) $$\mathrm{sgn}(\chi )=\{\begin{array}{cc} 1 & \chi =1\\ 0 & else \end{array}$$

so that:

(11) $$N\left(X\right)=\overset{N_{U}}{\underset{i=1}{\sum }}\mathrm{sgn}(\overset{N_{T}}{\underset{j=1}{\sum }}\chi_{ij}^{kp})$$

If the value of sgn(χ) is kept within the range of 0 to 1, the following condition is satisfied:

(12) $$\lambda =\;\{\begin{array}{c} 1,\;S\left(X\right)<1\\ 0,\;S\left(X\right)=1 \end{array}$$

Then, it can be ensured that the PI optimizes the maximum number of tasks, and after all the tasks are successfully executed, the number of UAVs is minimized at the same time. Since $\;N\left(X\right)\leq N_{U}$, the following must hold:

(13) $$J_{1}\left(X_{1}\right)=\;N\left(X_{1}\right)<J_{1}\left(X_{2}\right)=N_{U}+2-S\left(X_{2}\right)$$

This equation is a universal calculation formula for all the possible decision variables X. Therefore, when designing the task weight coefficient *w*, it is necessary to satisfy the following requirement:

(14) $$\overset{N_{T}}{\underset{j=1}{\sum }}w_{j}=1$$

Denote $w=\left[w_{1},w_{2},w_{3},\ldots ,w_{N_{T}}\right]$, and $w^{\prime }=\left[0,w\right]$ represents the extension vector of a weight vector. Then, there is the following expression:

(15) $$S\left(X\right)=\sum_{k=1}^{N_{k}}\sum_{p=1}^{N_{p}}\chi^{kp}{w^{\prime }}^{T}\leq 1$$

If and only if the non-zero row element of the matrix of all elements in decision variable X is *N_T_*, then all targets have been successfully reconnoitered.

(2) PI based on flight distance:

(16) $$\min J_{2}\left(X\right)=\overset{N_{s}}{\underset{k=1}{\sum }}\overset{N_{p}}{\underset{p=1}{\sum }}\underset{i\in T}{\sum }\underset{j\in T}{\sum }Dis_{ij}\chi_{ij}^{kp}$$

where $Dis_{ij}$ represents the distance from node *i* to node *j*. The total optimization PI can be written as follows:

(17) $$minJ\left(X\right)=f\left(J_{1}\left(X\right),J_{2}\left(X\right)\right)$$

The PI must satisfy the following constraints [29]:

(a) Each target is scouted for only once:
  (18) $$\overset{N_{s}}{\underset{k=1}{\sum }}\overset{N_{p}}{\underset{p=1}{\sum }}\underset{i\in T}{\sum }\underset{j\in T}{\sum }\chi_{ij}^{kp}-1\leq 0$$
(b) The number of UAVs cannot exceed the maximum number in the base:
  (19) $$\overset{N_{p}}{\underset{p=1}{\sum }}\mathrm{sgn}(\overset{N_{T}}{\underset{j=1}{\sum }}\chi_{ij}^{kp})-N_{k}\leq 0$$
(c) Each UAV departs from the base and eventually returns to the base (as mentioned above, the base is noted as 0):
  $\forall j=1,2,3,\ldots ,\;N_{T}$, and since $\chi_{ij}^{kp}=1$, then:
  (20) $$\{\begin{array}{c} \overset{N_{T}}{\underset{l=1}{\sum }}x_{0l}^{kp}=1\\ \overset{N_{T}}{\underset{l=1}{\sum }}x_{l0}^{kp}=1 \end{array}$$
(d) Time window constraints:
  Define the time window of task *j* $:\left[ms_{j},mo_{j}\right]$, $T_{j}$ indicates when the UAV will detect the target; therefore, the constraint can be given as follows:
  (21) $$ms_{j}\leq T_{j}\leq mo_{j}$$
  (22) $$T_{j}=\;T_{i}+Ser_{i}^{k}+t_{ij}^{k}+t_{C}{}_{j}$$
  where $Ser_{i}^{k}\;$ is the reconnaissance time of the *kth* type of UAV, $t_{ij}^{k}$ indicates the time for the *kth* type of UAV to fly from node *i* to node *j*, and $t_{Cj}$ is the wait time before executing the task.
(e) Sensor constraints of tasks:
  The minimum sensor accuracy required for the task *j* is $\;SenT_{j}$, the sensor accuracy of the *kth* type of UAV is $\;SenV_{j}^{k}$, and the constraint can be given as follows:
  (23) $$SenV_{j}^{k}-SenT_{j}\leq 0$$
(f) The total flight time of each UAV cannot exceed the maximum flight time:
  (24) $$\overset{N_{T}}{\underset{i=0}{\sum }}\overset{N_{T}}{\underset{j=1}{\sum }}\chi_{ij}^{kp}\left(t_{ij}^{k}+t_{C}{}_{j}+Ser_{j}^{k}\right)+x_{last-0}^{kp}t_{last-0}^{k}-TL_{k}\leq 0$$
  where node 0 represents the base by default, *last* represents the last reconnaissance task point of the UAV, and the latter item $t_{last-0}^{k}$ represents the time to return from the last task point.

## 3. Modified SOS for MTWDTSP

The original MOSOS algorithm works on the cooperative behavior seen among organisms in nature. During the search process, each organism benefits from continuous interaction with others in three different phases [24]: mutualism, commensalism and parasitism. Mutualism allows the two sides to benefit from each other; commensalism benefits one party, while the other party is not affected; parasitism benefits one party, and the other party suffers. These three phases are adopted from the most common symbioses used by organisms to increase their fitness and survival advantage over the long term. The mechanisms for updating the best organism are triggered after one generation of organisms has completed its three phases. The phases are repeated until the stopping criterion is achieved. The original MOSOS enjoys some advantages such as simplicity in operation, few control parameters, good stability, and strong optimization ability, but there are several shortcomings such as early maturity and delay in later-search [30]. As for the proposed problem, combining with the motivation to improve the diversity and quality of the solution, the accuracy of the algorithm convergence, and reduce the computational complexity, several modifications are introduced to the original MOSOS algorithm [31]. A “Pareto dominance determination” approach [32] is adopted to preprocess the performance indicator of the proposed problem to improve the diversity of non-inferior solutions. Meanwhile, globally adaptive approach is used to improve speed, accuracy and convergence characteristics of the original MOSOS.

### 3.1. Double-Chain Encoding of the Decision Variable

Considering the constraints of the multi-UAV reconnaissance task assignment problem, a double-chain encoding method is developed, where both chromosomes are encoded by integers. In Section 2, the decision variable is given by Formulas (4)–(6); and then the individual variable dimensions for each particle are as follows:

(25) $$N_{p}\times N_{k}\times T_{max}\times T_{max}$$

Considering the dimension, the number of individuals and the iteration equations, undoubtedly, there will be a complication of the problem and a slow convergence of the algorithm. As noted above, each element of the decision variable χ is in the form of a sparse matrix. If the sparse matrix is compressed and the location of nonzero elements is stored, then the particle’s variable dimension will be compressed as follows:

(26) $$N_{p}\times N_{k}\times M\times 2\times 2$$

where *M* represents the number of nonzero elements in each sparse matrix. Compared to the original coding, the decision space is greatly compressed, but it is still large. It can be imagined that there will still be a large number of zero matrices in the optimal decision variable χ.

Considering the constraint functions (a), (b) and (c) proposed in Section 2.4, the double-chain encoding is used from the perspective of the task point, and the decision code string is then mapped to the task sequence of each UAV. For example, for the assignment of six tasks, there are currently three types of UAVs, each of which has 3, 2, and 3 UAVs, so that if *T_UAV_ = (i,j)* represents the *jth* drone of the *i*th type, the example can be easily constructed as in Table 1.It is clear that for the UAV (1,1), the UAV passes through mission points 3 and 5, with mission point 5 flown to first and mission point 3 flown to last. A detailed decoding table is constructed as shown in Table 2.

Considering the uniform dimensions of the double-chain encoding, *T_UAV_ = (i,j)* is reduced dimensionally so that the following formulas are satisfied:

(27a) $$\mathrm{If}\;N=\overset{N_{k}}{\underset{k=1}{\sum }}n_{k}\;\mathrm{and}\;{\left\|T_{UAV}=\left(i,j\right)\right\|}_{1}=M,$$

(27b) $$\mathrm{then}\;\{\begin{array}{c} i=N_{p}\\ j=n_{N_{k}}-\left(\overset{N_{p}}{\underset{p=1}{\sum }}n_{p}-M\right)\\ \overset{N_{p}-1}{\underset{p=1}{\sum }}n_{p}<M\leq \overset{N_{p}}{\underset{p=1}{\sum }}n_{p} \end{array}$$

In summary, the decision variables of $2\times N_{T}$ dimension can be defined to describe the mission assignment status of the UAV, and $N_{T}$ represents the number of tasks. The assignment scheme generated by this encoding determines that each task point is visited by a UAV, but in reality, due to constraints such as the time window, there will be situations where certain task points cannot be executed. M can be set to zero to indicate that a UAV was not available to execute the task.

### 3.2. Pareto Dominance Determination and Optimal Solution Set

According to the mathematical model constructed in Section 2, the task assignment problem can be summarized as a nonlinear multi-objective optimization (minimum) problem:

(28) $$\begin{array}{c} minf(X)=(f_{1}(X),f_{2}(X),f_{3}(X)\ldots f_{n}(X))\\ \begin{array}{cc} X\in \Omega \in R^{n} & s.t\;c_{i}(X)\leq 0 \end{array} \end{array}$$

where $X=\left(\chi_{1},\chi_{2},\chi_{3},\ldots ,\chi_{n}\right)$ is the *n*-dimensional decision vector in $R^{n}$ space and $c_{i}\left(X\right)\leq 0$ (i=1,2,3,…,p) represents the constraints of the optimization problem. The series of sub-PIs may conflict with each other; this nonlinear multi-objective optimization problem will produce multiple solutions. French economist V. Pareto proposed the concept of Pareto solution set for comparing the different feasible solutions. Assuming that for the minimization optimization problem, there are two feasible solutions $X_{1}\;$ and $\;X_{2}$, if the following holds:

(29) $$\forall f_{i}\left(X\right),f_{i}\left(X_{1}\right)\leq f_{i}\left(X_{2}\right)\;\mathrm{and}\;\exists J_{k}\left(X\right),f_{i}\left(X_{1}\right)<f_{i}\left(X_{2}\right)$$

**Figure 6:**
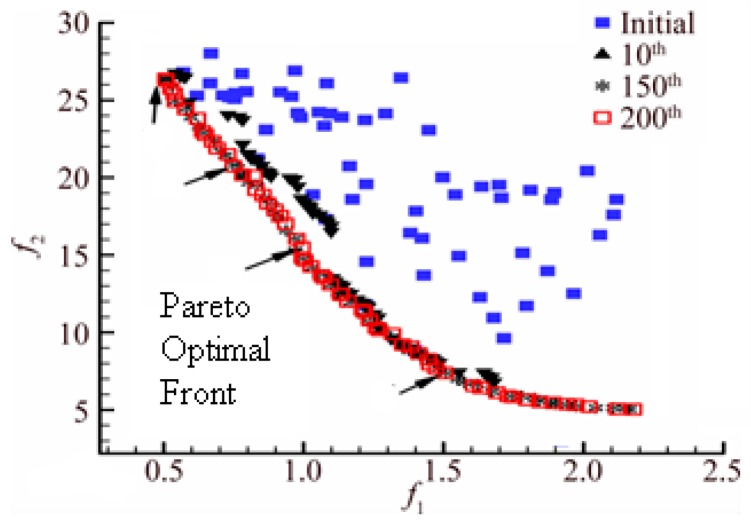
Distribution of Pareto optimal front.

Then, $X_{1\;}$ dominates $\;X_{2}$, and $\;X_{1}$ is called a noninferior solution, and $\;X_{2}\;$ is called an inferior solution. If solutions $\;X_{1}\;$ and $X_{2}$ are not dominated by each other, then both solutions are noninferior solutions and are added to the noninferior solution set. This solution set is the so-called “Pareto solution set” and is often referred to as the optimal frontier (as shown in Figure 6). 

Figure 6 illustrates the process of minimizing the optimization of the two indicator functions. It can be clearly seen that as the number of iterations increases, the distribution of the optimal solution set tends to increasingly form a curve, and the red squares indicate the best frontier after 200 iterations. Then, using the artificial knowledge, the feasible solutions in the set are analyzed and compared based on different weights of PIs.

This paper focuses on the assignment problem for UAVs with obstacles and threats in the flight zone and presents a modified SOS algorithm based on Pareto dominance determination inspired by traditional evolutional algorithms and PSO. At the same time, a suitable coding operator is designed to effectively address all types of constraints to solve the task assignment problem.

### 3.3. Modified SOS with Global Adaptive Scaling Factor

Specific steps of the SOS algorithm can be expressed as follows:

*Step 1*. Initialize the population: First, generate *N_b_* random individual “biologicals” according to Equation (30); each "biological" is an initial solution.

(30) $$X_{i}=l_{b}+rand(1,D_{Χ})\cdot (u_{b}-l_{b})$$

where $X_{i}$ represents the *i*th (*i = 1, 2,..., N_b_*) "biological" in the ecosystem, *D_X_* is the dimension of the solution, and *rand(1,D_X_)* is a *1 × D_X_* dimension scaling factor vector. *u_b_* and *l_b_* are upper and lower bounds of the search space, respectively.

*Step 2.* Mutualism: At this stage a “biological” *X_j_* is picked randomly from the population to interact with *X_i_* and produce mutual benefits so that each progresses toward the optimal solution. *X_i_* and *X_j_* generate new solutions of *X_i_* and *X_j_* according to Equation (31). In the formula, *X_best_* is the optimal individual in the ecosystem, *V_M_* is a symbiotic vector and represents the middle point of the two individuals, and *BF* is the benefit factor; since each individual has a different degree of benefit, *BF* takes a value of 1 or 2.

(31) $$\begin{array}{l} X_{inew}=X_{i}+rand(0,1)\cdot (X_{best}-V_{M}\cdot BF_{1})\\ X_{jnew}=X_{j}+rand(0,1)\cdot (X_{best}-V_{M}\cdot BF_{2})\\ V_{M}=\frac{X_{i}+X_{j}}{2} \end{array}$$

*Step 3. Globally Adaptive Parasitism* [31]: For the original MOSOS algorithm, only one individual can benefit in a symbiotic relationship, while another individual is not affected. For the *ith* individual $\;X_{i}$, one individual $X_{j}\;$ is randomly selected from other individuals. The original MOSOS algorithm always uses the search strategy of “current-to-best.” Because of the randomness of the scaling factor, the search equation limits the guiding effect of the global optimal value, resulting in insufficient convergence accuracy and a long convergence time, which does not meet the requirement of the task assignment problem. For this reason, this paper proposes an adaptive hybrid search strategy with global optimal guidance. The specific form is as follows:

(32a) $$X_{inew}=X_{i}+(1-\mu )(X_{i}-X_{j})+\mu (X_{best}-X_{j})$$

(32b) $$\mu =\mu_{\min}+(\mu_{\max}-\mu_{\min})\frac{G_{I}}{G_{\max}}$$

where μ∈[0,1] is the scaling factor; $\mu_{\max}$, $\mu_{\min}$ are the maximum and minimum scaling factors, respectively; *G_max_* is the maximum number of cycles; and *G_I_* is the current number of iterations. This strategy maintains the difference between individuals by adding a differential disturbance factor. It simultaneously introduces an adaptive scaling factor. The initial cycle has a smaller μ. The algorithm emphasizes the global search, reduces the tendency to move toward a single point in the search space, and prevents the algorithm from falling into a local minimum. With the progress of the algorithm, μ gradually increases, the guiding role of the current optimal individual is strengthened, the leading algorithm advances, and the search process is no longer just a complete random search but instead is more purposeful and directional.

### 3.4. Optimal Solution Selection of the Proposed Algorithm 

The core of the MOSOS algorithm is to determine the global optimal solution $\;X_{best\;}$ according to Pareto dominance determination, and only one set of noninferior solutions can be identified. Since the optimal solution must exist in the noninferior solution set, in this study the external noninferior profile ***C*** of the “biological” population is constructed for the selection of global optimal solution $\;X_{best\;}$. Refer to the selection technique used in [29], solutions in the noninferior solution set is sorted by their dominance firstly, then compared with the external noninferior profile ***C***, and replace the dominated solutions in profile ***C*** with better ones. 

At the meantime, since each iteration of the MOSOS algorithm will inevitably generate many new solutions, the scale of external non-inferior profile will increase dramatically. With the purpose to distribute the optimal front edge as evenly as possible, it is necessary to eliminate a part of the densely distributed individuals and avoid falling into local optimum. Therefore, the external noninferior profile need to be “pruned” during the iterations. Refer to the crowded function defined in NSGA-II, an individual density function for sorting the solutions with normalized PIs and constraints is developed in this study as follows:

(33) $$Den_{i}=\frac{1}{{\left\|\left(J_{i+1,1}-J_{i-1,1}\right)\right\|}_{2}+{\left\|\left(J_{i+1,2}-J_{i-1,2}\right)\right\|}_{2}}$$

**Figure 7:**
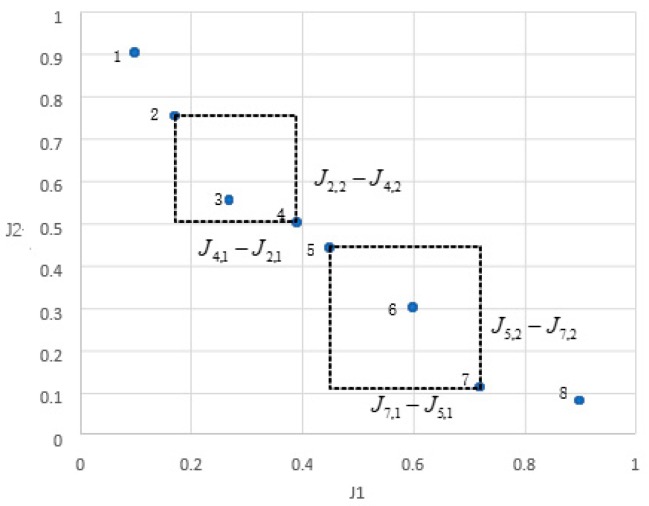
Density function between individuals.

As shown in Figure 7, ${\left\|\left(J_{i+1,1}-J_{i-1,1}\right)\right\|}_{2},{\left\|\left(J_{i+1,2}-J_{i-1,2}\right)\right\|}_{2}$ are the length and width of the quadrilateral, and the density of the individual *i* is reciprocal of their sum. Which reflects, the larger ${\left\|\left(J_{i+1,1}-J_{i-1,1}\right)\right\|}_{2},{\left\|\left(J_{i+1,2}-J_{i-1,2}\right)\right\|}_{2}$, the lower density of individual *i.*

Considering the efficiency of the proposed algorithm, the size of the external non-inferior profile ***C*** is limited to less than 1/5 of the size of the ecosystem. The following operations are required during the iterations of MOSOS: when the number of non-inferior solutions in the profile ***C*** exceeds its maximum size, calculate the density of each non-inferior solution. Then eliminate the individuals with the highest density until the number of non-inferior solutions does not exceed the largest scale of profile ***C***. 

In the end, in order to let individuals to track the position of *X_best_* quickly and accelerate the convergence of the algorithm, the individual with the lowest density in the external non-inferior profile ***C*** is selected as the global optimal solution *X_best_*. The algorithm of optimal solution selection is given as follows:

**Algorithm 1:** Algorithm of the optimal solution selection.
Step 1.Initialize all the “biologicals” individual variables and calculate their PI values and constraint function values.Step 2.According to Section 3.1, all optimal noninferior solutions are added to the external nontrivial file *C*.Step 3.Execute the SOS search iteration formula to obtain a new “biologicals” individual X and calculate its index function value and constraint function value.Step 4.Compare all the solutions in the new X with the external noninferior files *C* according to the criterion. If the new solution dominates the solution in *C*, then the inferior solution in *C* is deleted and the new solution is added to *C*; if the new solution and the solution in *C* do not dominate each other, the new solution is also added to *C*; if the new solution is dominated by the *C* solution, nothing is done.Step 5.Conduct pruning operations on the non-inferiority external profile *C*, and select *X_best_*.Step 6.Repeat Steps 2–5 until the SOS algorithm reaches the maximum number of iterations.


## 5. Numerical Simulation Results and Analysis 

The proposed method for task assignments was tested via numerical simulation experiments. The numerical experiments were created by using Visual C++ (Ver. 10.0), and the figures illustrating the results were created using MATLAB. The entire simulation was performed on a workstation consisting of a 3.5 GHz Intel Core-i7 CPU and 16 GB of physical RAM running 64-bit Microsoft Windows 10.

A total of 12 tasks were set in the battlefield. The parameter settings for all task points are shown in Table 3. The minimum turning radius *r_min_* is set as 1 km, and the detection width *W_U_* is set as 2 km.

**Figure 8:**
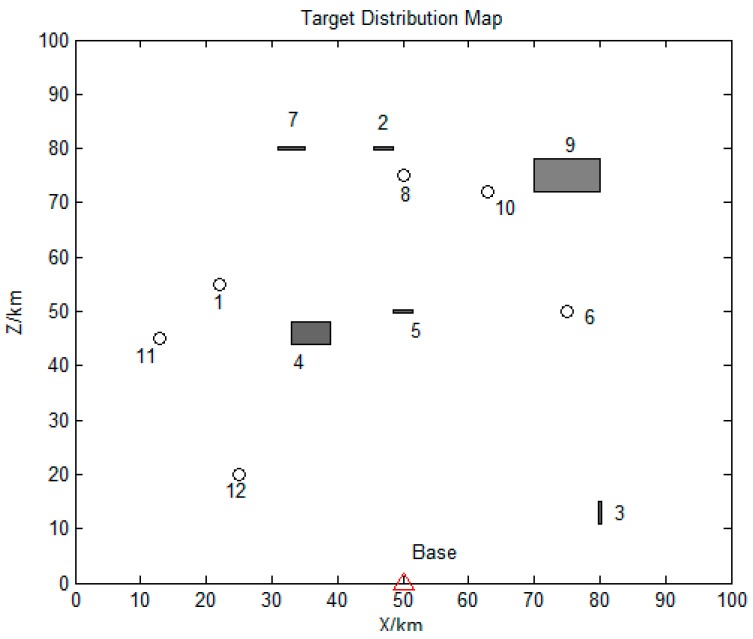
Distribution map of all task points.

The distribution map of all task points is shown in Figure 8. Circles represent for the point targets, short strips and rectangles stand for strip targets and surface targets, respectively.

There are the categories of UAVs on the battlefield, located at the starting positions (50, 0), of which parameters are shown in Table 4.

The MOSOS algorithm is set to have a population size of 30; the number of termination iterations *G_max_* is 200, with a maximum external profile size of 30 and an external file of “biological” individual size of 5, scaling factors $\mu_{\max}=0.87$ and $\mu_{\min}=0.13$. 

**Figure 9:**
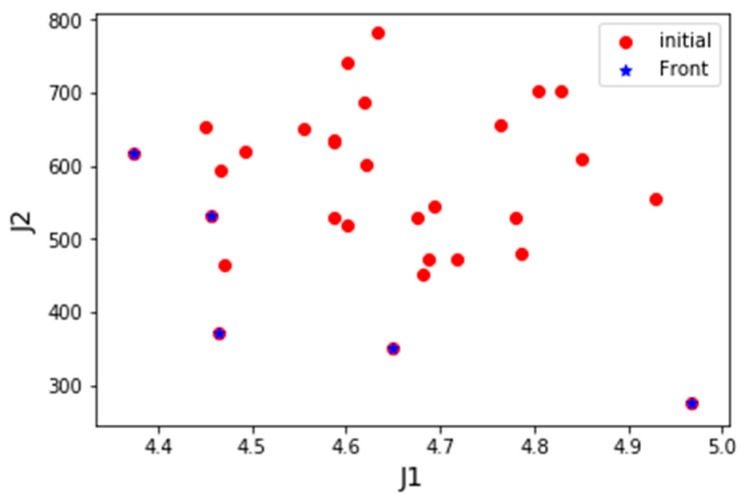
Initial “biological” performance index distribution of Scenario 1.

**Figure 10:**
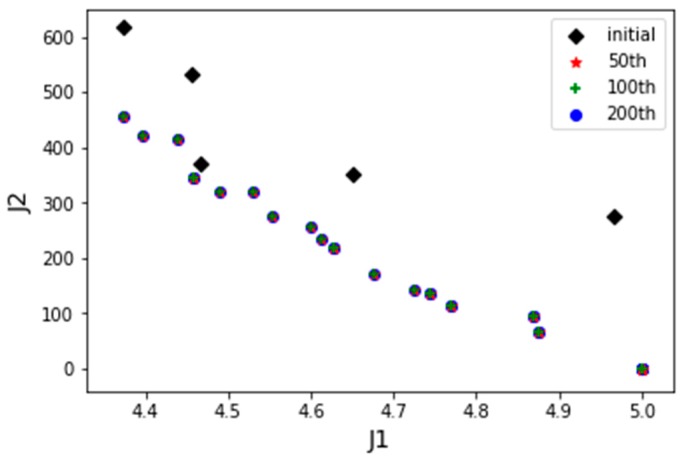
The best frontier distribution in history of Scenario 1.

*Scenario 1*: One UAV of each type, with a total of 3 UAVs are employed in the base for task assignment. Since the number of UAVs is much smaller than the number of tasks, UAVs are unable to complete of the tasks, then the adjustment parameter λ is set to 1 in this scenario. Figure 9 displayed the distribution of PIs of the initial positions of the population and the optimal frontier distribution of the initial generation. Figure 10 shows the evolution of the history frontier distribution in the iterative process, including the initial generation, the 50th generation, the 100th generation, and the 200th generation.

From the perspective of implementing tasks more economically in the real combat, it is expected that existing UAVs could implement the tasks as much as possible, or implement all the tasks with less UAVs. Thus, the final feasible solution is selected as the one with the smallest value of PI J1, then code of assignment variables to the task is constructed as follows:

$$\left[\begin{array}{cccccccccccc} 0 & 0 & 0 & 1 & 1 & 0 & 3 & 2 & 2 & 0 & 0 & 3\\ 1 & 1 & 1 & 2 & 1 & 1 & 1 & 2 & 1 & 1 & 1 & 2 \end{array}\right]$$

**Figure 11:**
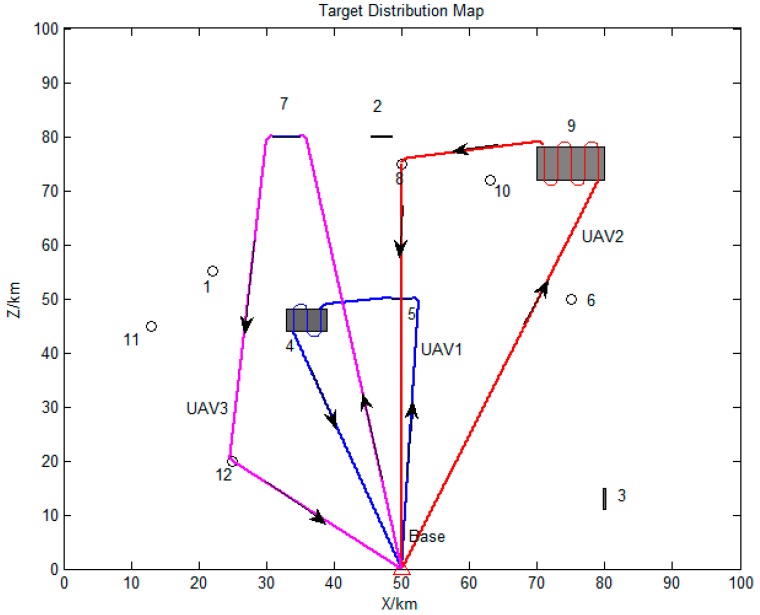
Paths of all UAVs in Scenario 1.

The serial number and detailed flight sequence of each UAV is shown in Table 5, while the paths of each employed UAV are displayed in Figure 11. It can be seen that all the UAVs are employed to execute tasks with significant weight under permitted conditions. Besides, the reconnaissance sequences of the assigned targets for the UAVs are in a good order, and the total flight distance is 456.19 km. Thus, proposed method can obtain satisfied task allocation results for multi-UAV cooperative reconnaissance problems on heterogeneous targets. 

**Figure 12:**
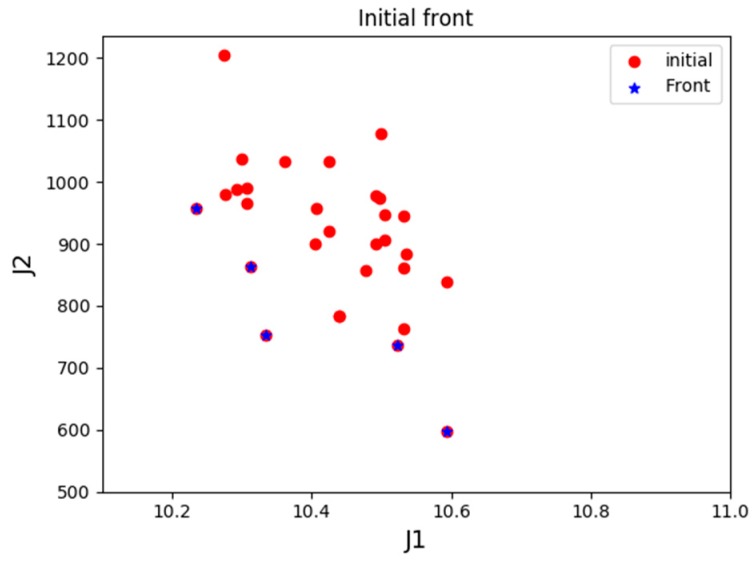
Initial “biological” performance index distribution in Scenario 2.

*Scenario 2*: Three UAVs of each type, with a total of 9 UAVs are prepared in the base for task assignment. Figure 12 displayed the distribution of PIs of the initial positions of the population and the optimal frontier distribution of the initial generation. 

**Figure 13:**
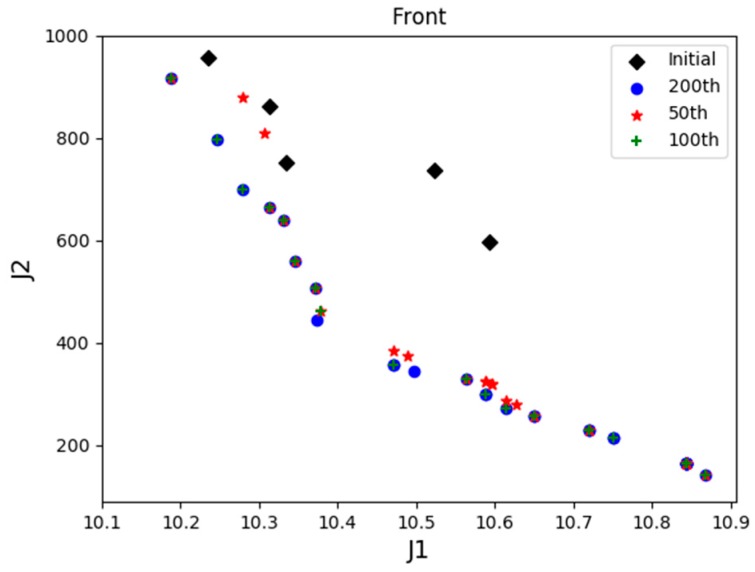
The best frontier distribution in history in Scenario 2.

Figure 13 shows the evolution of the history frontier distribution in the iterative process, each point in the graph represents the best non-inferior solution to the number of iterations. Since there exists solutions for UAVs to complete all the tasks, the adjustment parameter λ is set to 0 in the latter stage of optimization. As seen in the figure, with the increase in the number of iterations of the algorithm, the external archive is continuously approaching the best frontier, indicating that the algorithm is convergent. The front-end distribution of all generations of noninferior solutions is uniform, which reflects the distribution of the real optimal frontier. 

If taking the most reconnaissance tasks completed as the main PI, that is, the index function *J_1_*, then in this simulation process, a total of 6 drones were deployed to perform reconnaissance tasks of all 9 UAVs, and a total of 12 task points were visited. The code of assignment variables to the task is constructed as follows:

$$\left[\begin{array}{cccccccccccc} 2 & 1 & 5 & 2 & 6 & 5 & 9 & 2 & 3 & 1 & 6 & 5\\ 2 & 2 & 1 & 1 & 1 & 2 & 1 & 3 & 1 & 1 & 2 & 3 \end{array}\right]$$

**Figure 14:**
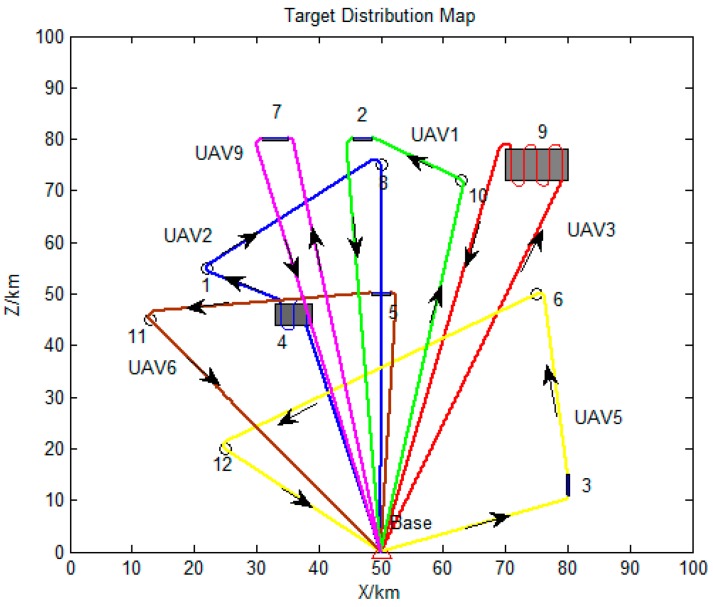
Paths of employed UAVs.

The detailed flight sequence of each UAV is shown in Table 6, while the paths of each employed UAV are displayed in Figure 14. It can be seen that all the targets are visited by the employed UAVs. The total flight distance of all employed UAVs is 937.52 km. Thus, it is proved once again that the proposed method can obtain satisfied task allocation results for multi-UAV cooperative reconnaissance problems on heterogeneous targets.

With the intention to verify the superiority of the proposed method (IMOSOS) in Section 3, improved MOPSO (IMOPSO) [33], NSGA-II [12] and original MOSOS with the same simulation conditions and stopping criterion are implemented for comparison. The initial parameters of IMOPSO are set as: *w_max_* = 0.9; *w_min_* = 0.4; factor *C*1 = *C*2 = 1.454. The initial parameters of NSGA-II are set as: probabilities of crossover and mutation are 0.9 and 0.1, respectively; weight factors *α* and *β* of sub-objectives are both set to be 0.5. The initial parameters of original MOSOS is the same as the proposed method. The final feasible solutions of all the algorithms are selected as the one with the smallest value of PI *J*_1_.

Monte-Carlo simulations on Scenario 2 are carried out, as the 12 tasks mentioned above with random positions. And the statistical results of 200 runs displayed in Table 7. Among the results are the best, worst, and average (i.e. Avg.) value of the PIs proposed in Section 3. Also, the compute efficiency of each algorithm is given in Table 7. Table 8 shows the improvement ratio of IMOSOS compared with other algorithms. From the statistical results of the referred indicators, the IMOSOS performs better in searching optimal solutions to the certain task assignment problem than the other three algorithms. Meanwhile, according to the average results of the referred indicators, the improved MOSOS can provide stable solutions as the three algorithms. Thus, with the help of problem formulation of MTWDTSP and modification to the original MOSOS, this proposed method enjoys certain superiority and efficiency for multi-UAV reconnaissance task allocation problems with heterogeneous targets.

Three main aspects have considerable influence on the computational efficiency of the algorithm in the simulation process: the number of “biological” individual, the iterations of the algorithm and the scale of the external non-inferior archive. The increased number of task points and the number of the UAVs have little effect on the computational efficiency of the algorithm. The encoding rules ensure that the number of task points is linearly related to the calculation time of the algorithm. If more tasks, UAVs or bases are considered in this particular problem, in order to solve the problem effectively, the size of encoded decision variables and iterations may increase correspondingly. Therefore, for the problem of multi-UAV multi-reconnaissance tasks, the proposed method can provide a valid suboptimal solution.

## 6. Conclusions

This paper considers a reconnaissance task assignment problem for multi-UAVs as a multi-objective, multi-constraint nonlinear optimization problem. A model of cooperative UAVs and reconnaissance tasks of heterogeneous ground targets with time windows is built to describe this problem. To solve the task assignment problem, two PIs were constructed and need to be optimized. When dealing with the multi-UAV task assignment optimization model, this paper adopts the modified MOSOS algorithm based on the Pareto dominance determination and the global adaptive operation. To address the various constraints of the task assignment model, the decision variables designed from each UAV itself are mapped to the task point of view. Therefore, the double-chain encoding for modified MOSOS inherently meets the constraint requirements of the task assignment model and greatly reduces the dimensionality of the decision variables, thereby speeding up the convergence of the algorithm. The global best external archive and the best external individual archive are designed in this study. Furthermore, this study defines the density function of each individual, thus ensuring that the solution to the problem meets the requirements of diversity and homogeneity. Finally, numerical simulation results and statistical results of Monte-Carlo simulations are proposed to verify the superiority and efficiency of the introduced method.

## Figures and Tables

**Table 1:** Example of UAV Task Assignment.

Task Number	1	2	3	4	5	6
*T_UAV_*	(2,1)	(3,2)	(1,1)	(2,1)	(1,1)	(3,1)
Task Order	1	1	2	2	1	1

**Table 2:** Task Assignment Decoding.

T_UAV_	(1,1)	(2,1)	(3,1)	(3,2)
Task Timing	5-3	1-4	6	2

**Table 3:** Parameters of Task Point Parameters.

Task No.	Position/km	Time Win./s	Sensor Req.	Shape	Size/km	Weight Para.
1	(22,55)	(100,300)	3	Point	-	0.04587509
2	(47,80)	(100,200)	3	Strip	4.2	0.10454398
3	(80,13)	(100,200)	3	Strip	5.1	0.12473234
4	(36,46)	(250,500)	2	Surface	6*2	0.13173999
5	(50,50)	(250,500)	2	Strip	4.0	0.09815578
6	(75,50)	(250,500)	2	Point	-	0.03243373
7	(33,75)	(750,1000)	1	Strip	5.6	0.11667218
8	(50,75)	(750,1000)	1	Point	-	0.09324776
9	(75,75)	(650,1000)	1	Surface	8*4	0.15580116
10	(63,72)	(500,750)	2	Point	-	0.02624827
11	(13,45)	(1100,1300)	2	Point	-	0.03795899
12	(25,20)	(560,1500)	2	Point	-	0.03259074

**Table 4:** Parameters of UAVs.

Category	Max. Speed	Max. Cru. Time	Sensor Level
1	230 m/s	800 s	3
2	310 m/s	420 s	3
3	170 m/s	1100 s	2

**Table 5:** Flight Sequence of Each UAV in Scenario 1.

UAV No.	UAV Category	Task Sequence
1	1	5-4
2	2	9-8
3	3	7-12

**Table 6:** Flight Sequence of Each UAV in Scenario 2.

UAV No.	UAV Category	Task Sequence
1	1	10-2
2	1	4-1-8
3	1	9
5	2	3-6-12
6	2	5-11
9	3	7

**Table 7:** Statistical results of Monte-Carlo simulations on Scenario 2.

Indicator	J1			J2 (km)			Compute Efficiency (s)		
Algorithm	Best	Worst	Avg.	Best	Worst	Avg.	Best	Worst	Avg.
IMOSOS	6	8	6.47	937.52	1089.71	956.45	9.2	17.5	12.7
MOSOS	6	9	7.33	941.27	1146.49	1017.92	14.3	22.9	17.1
NSGA-II	6	9	7.21	932.31	1123.56	998.41	16.4	26.6	21.3
IMOPSO	6	8	7.21	939.26	1109.56	979.9	12.6	20.9	15.3

**Table 8:** Improvement ratio of IMOSOS compared with other algorithms.

Indicator	J1	J2	Compute Efficiency
MOSOS	11.7%	6.1%	25.7%
NSGA-II	10.2%	4.2%	40.2%
IMOPSO	10.2%	2.0%	16.9%

## Data Availability

The values of some of the simulation parameters are available online (literatures), and other data is original from the authors of this paper.

## Abbreviations

*x*	The UAV’s coordinates in plane space
*z*	The UAV’s coordinates in plane space
*V_U_*	Speed of the UAV
*r_min_*	Minimum turning radius of the UAV
ψ	The flight yaw angle of the UAV
*H_U_*	The flight altitude of the UAV
*W_U_*	The detection width of the sensor
*t_d_*	Target detection time
*t_Dec_*	Detection time of the point target
*L_st_*	Extension length of the strip target
*L_sf_*	Length of the smallest circumscribed rectangle
*W_sf_*	Width of the smallest circumscribed rectangle
*T_Task_*	Time window of the task
$t_{begin}$	The earliest time available for detection
$t_{end}$	The latest time available for detection
$t_{c}$	The waiting time before execution
$N_{U}$	Number of UAVs
$N_{T}$	Number of reconnaissance tasks
${Χ|}_{N_{U}}^{N_{T}}$	The decision variable
$J_{Asn}$	Performance indicator of the task assignment problem
$C_{Asn}$	Constraint of the task assignment problem
$\chi_{ij}^{kp}$	Element of the decision variable
**T**	The set of the number for task points
*N_k_*	The number of types of the UAV
*N_p_*	The number of the UAV of the *kth* type
$J_{1}$	Performance indicator of tasks/UAVs
λ	Adjustment parameter
S(Χ)	The total weight of the tasks that have been assigned
N(X)	The number of the employed UAVs to execute the assigned tasks
sgn(⋅)	Symbol function
w	Weight vector
$J_{2}$	Performance indicator of flight distance
$Dis_{ij}$	The distance from task *i* to task *j*
$SenT_{j}$	The minimum sensor accuracy required for the task *j*
$SenV_{j}^{k}$	The sensor accuracy of the *kth* type of UAV
$X_{i}$	The *ith* (*i = 1, 2,..., N_b_*) "biological" in the ecosystem
*D_X_*	The dimension of the solution
BF	The benefit factor
μ	The scaling factor
*G_max_*	The maximum number of cycles
*G_I_*	The current number of iterations
***C***	The external noninferior profile of the noninferior solution set
